# Microbial cues and T cell immunity: deciphering their interactions in chronic liver disease

**DOI:** 10.1038/s12276-026-01831-y

**Published:** 2026-10-09

**Authors:** Yinuo Liu, Baichao Yu, Qiaoyan Liu, Xiong Ma, Ruqi Tang

**Affiliations:** NHC Key Laboratory of Digestive Diseases (Renji Hospital, Shanghai Jiao Tong University School of Medicine), Division of Gastroenterology and Hepatology, State Key Laboratory for Oncogenes and Related Genes, Renji Hospital, School of Medicine, Shanghai Jiao Tong University; Shanghai Institute of Digestive Disease, 145 Middle Shandong Road, Shanghai, 200001 China

**Keywords:** Lymphocyte activation, Liver diseases

## Abstract

Chronic liver disease (CLD) poses a major global health challenge, as its progression to cirrhosis and hepatocellular carcinoma significantly contributes to worldwide morbidity and mortality. Mounting evidence has identified the gut microbiota as a critical modulator of CLD progression, primarily via the gut–liver axis. The liver is continuously exposed to gut-derived signals through the portal circulation, while reciprocally secreting bioactive mediators that shape intestinal microbial composition and function. This bidirectional network enables intricate crosstalk between the gut microbiota and the host immune system. In particular, T lymphocytes, the central effectors of adaptive immunity, are tightly regulated by gut microbiota and their metabolites. Conversely, T cells also modulate the gut microbial ecosystem. Herein, we summarize current knowledge on this bidirectional crosstalk, focusing on how gut microbiota shape T cell differentiation, function and plasticity, as well as the reciprocal impact of T cells on the gut microbiota. Furthermore, we evaluate how these interactions influence the development and progression of various CLDs, including autoimmune liver diseases, alcohol-associated liver disease, metabolic dysfunction-associated steatotic liver disease and viral hepatitis. Finally, we discuss the therapeutic potential of targeting gut microbiota–T cell interactions through immunomodulatory and microbiota-directed strategies to offer novel therapeutic approaches.

## Gut Microbiota and T Cells Shape Chronic Liver Disease Progression

Chronic liver disease (CLD) is a significant global health issue, causing millions of deaths annually, with limited therapeutic options. Recent research highlights the role of the gut microbiota in CLD pathogenesis, focusing on the gut–liver axis, where microbial products and immune responses, particularly T cells, influence liver health. This review explores how gut microbiota modulates T cell responses in CLD, including autoimmune liver diseases, alcohol-associated liver disease, metabolic dysfunction-associated steatotic liver disease and viral hepatitis. The authors discuss the interactions between gut microbiota and T cells, emphasizing the potential for novel therapeutic strategies targeting these interactions. Key findings include the modulation of T cell subsets by microbial metabolites, which can influence disease progression and immune responses. The review suggests that understanding these interactions could lead to innovative treatments for CLD.

This summary was initially drafted using artificial intelligence, then revised and fact-checked by the author.

## Introduction

Chronic liver disease (CLD) is a serious global health threat with multiple potential causes, including autoimmune liver diseases (AILD), alcohol-associated liver disease (ALD), metabolic dysfunction-associated steatotic liver disease (MASLD) and viral hepatitis^1^. The continuous progression of CLD leads to impaired liver function, causing the disease to develop into cirrhosis and hepatocellular carcinoma (HCC)^2^. CLD is reported to cause 2 million deaths worldwide each year and carries a significant disability burden. However, effective therapeutic options remain limited^3^. In this context, research on gut microbiota has brought new hope for the treatment of CLD.

The gut microbiota has increasingly been recognized as a critical contributor to CLD pathogenesis, largely because of the liver’s unique anatomical and functional relationship with the intestine. The liver is continuously exposed to microbial products and exogenous substances via the portal circulation, while simultaneously secreting bile acids (BAs) and other bioactive mediators that shape the intestinal microbial ecosystem. This reciprocal interaction establishes a bidirectional communication network, commonly referred to as the gut–liver axis^4,5^. Notably, the immune system is central to maintaining homeostasis within this axis, orchestrating a delicate balance between tolerance to commensal bacteria and robust immune defences against pathogens^6^. Among immune cells, T lymphocytes are garnering increasing attention as key regulators of gut–liver homeostasis, owing to their remarkable ability to recognize a wide spectrum of microbial antigens, and their functions are also profoundly influenced by microbial cues^7^. Emerging evidence indicates that gut microbiota–T cell interactions exert a significant influence on the onset and progression of liver diseases. For instance, our team recently demonstrated that treatment with the microbial metabolite indole-3-carboxaldehyde (ICA) can inhibit the activation of effector T cells, thereby ameliorating immune-mediated hepatitis^8^. Another study by Nakamoto et al. found that pathogenic bacteria derived from the faeces of patients with primary sclerosing cholangitis (PSC) aggravate hepatobiliary disease by inducing T helper 17 (Th17) responses in the liver^9^. Collectively, these findings highlight the importance of elucidating the crosstalk between gut microbiota and T cells, which may offer novel therapeutic strategies for the prevention and treatment of CLD.

In this review, we first introduce T cell responses to the gut microbiota and the role of gut microecology in CLD. Then we place emphasis on gut microbiota–T cell interactions in CLD, including AILD, ALD, MASLD and viral hepatitis. Finally, we propose new therapeutic insights with the hope of providing a future target for CLD.

## T cells modulated by gut microbiota

**Fig. 1:**
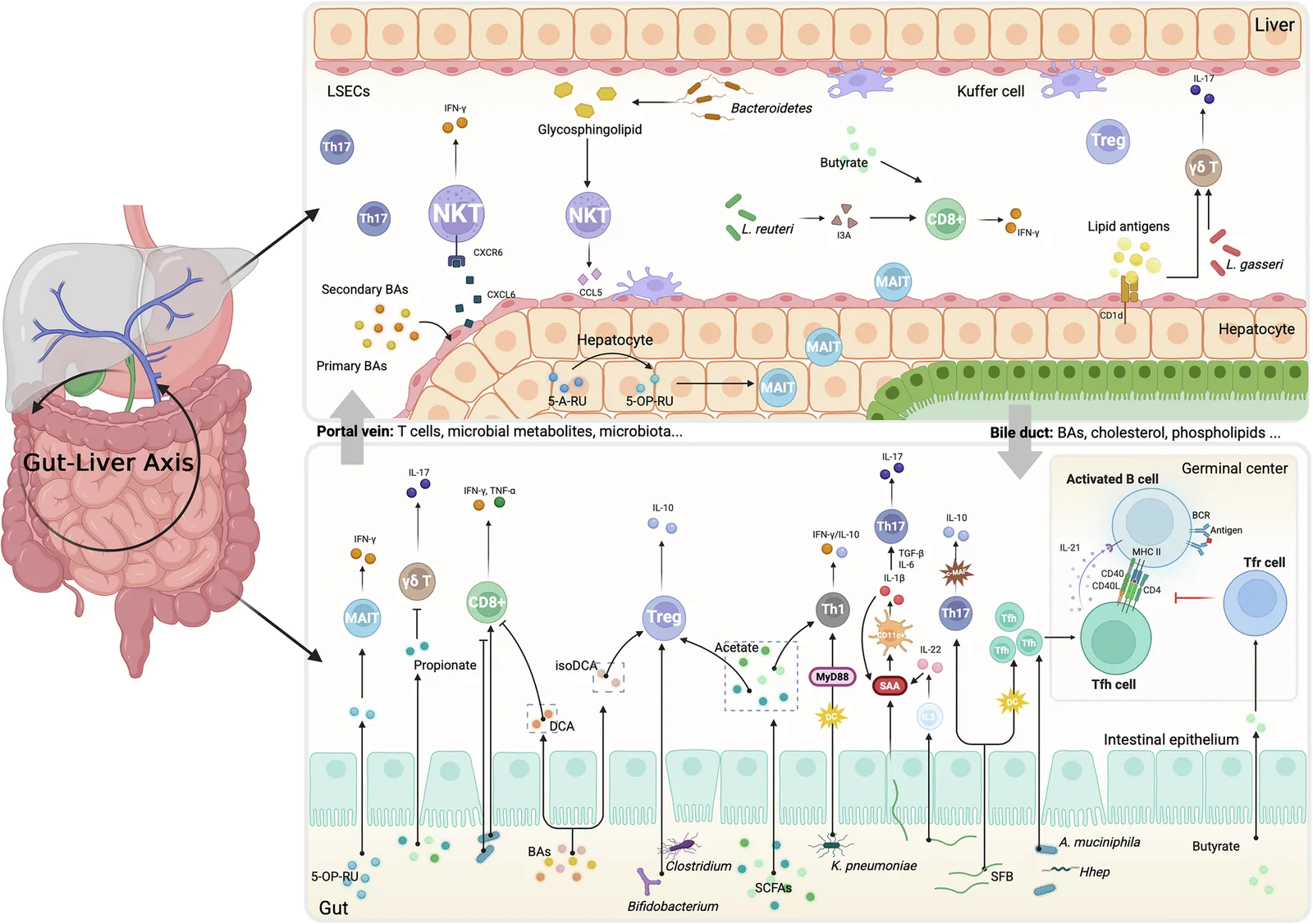
T cells regulated by microbiota and metabolites in the gut–liver axis. In the intestine, commensals (e.g. SFB, *A. muciniphila*, *K. pneumoniae*) and their metabolites (e.g. short-chain fatty acids (SCFAs), deoxycholic acid (DCA) etc.) direct the differentiation of key T helper subsets, including Th17, Th1, Tfh and Treg cells, and modulate CD8⁺ T cells, γδ T cells as well as MAIT cell function via intricate signalling pathways and feedback loops. These primed T cells, along with microbial components and metabolites, can translocate via the portal vein to the liver. Within the hepatic microenvironment, such gut-derived signals or intrahepatic bacteria further influence the function of resident and recruited T cells, such as NKT, MAIT, CD8^+^ T cells, and γδ T cell populations by modulating bile acid ratios and lipid antigen presentation. This continuous crosstalk establishes the gut–liver axis as a fundamental regulator of adaptive immunity. 5-A-RU, 5-amino-6-ribitylaminouracil; 5-OP–RU, 5-(2-oxopropylideneamino)-6-D-ribitylaminouracil; BA, bile acid; c-MAF, cellular musculoaponeurotic fibrosarcoma; DC, dendritic cell; EC, epithelial cell; IFN, interferon; IL, interleukin; I3A, indole-3-aldehyde; ILC3, type 3 innate lymphoid cells; isoDCA, 3β-hydroxydeoxycholic acid; LP, lamina propria; LSEC, liver sinusoidal endothelial cell; MyD88, myeloid differentiation primary response protein 88; PPs, Peyer’s patches; SAA, serum amyloid A; SFB, segmented filamentous bacteria; TGF, transforming growth factor; TNF, tumour necrosis factor.

Liver and intestine resident T cells are constitutively exposed to microbiota-derived antigens and their associated contexts. The gut microbiota-driven development, priming and functional modulation of these T cells are essential for sustaining host immune homeostasis, and the intricate interaction between these two compartments profoundly influences health and disease^10^. In this section, we discuss the biological roles of distinct T cell subsets within the gut–liver axis and how they can be selectively modulated by specific microbial taxa (Fig. 1).

### CD4^+^ T cells

#### Th17 cells

Th17 cells, as important mediators for intestinal host defence, regulate the immune balance between pathogen clearance and microbial colonization, which exemplifies the underlying host–commensal co-evolution^11^. Th17 cells are virtually absent in the intestinal lamina propria (LP) of germ-free (GF) mice, whereas their numbers increase following colonization with intestinal microorganisms or supplementation with microbial metabolites, highlighting the strict dependence of Th17 cells on microbial colonization^12,13^. Segmented filamentous bacteria (SFB), a commensal bacterium colonizing the ileum, represent a classical inducer of Th17 cells^14,15^. Mechanistically, SFB adhesion triggers intestinal epithelial cells to secrete serum amyloid A (SAA), which acts on CD11c^+^ cells to potentiate Th17 cell differentiation and effector function^16,17^. This effect is reported to be amplified by the SAA-interleukin (IL)-1β positive feedback loop. Specifically, SAA activates LP CD11c^+^ cells to produce IL-1β, which in turn promotes the expression of SAA in epithelial cells^14,17^. In this circuit, SFB upregulate the IL-22 produced by type 3 innate lymphoid cells (ILC3) as well, and IL-22 enhances the expression of SAA^16^. Previous studies have demonstrated that the colonization of SFB induces the production of IL-17 by host Th17 cells^14,16–18^. Recently, a study revealed that SFB-specific Th17 cells also acquire an anti-inflammatory phenotype characterized by IL-10 expression in a c-MAF-dependent manner to exert immunoregulatory functions that inhibit effector T cell activity^19^.

SFB colonization is difficult to detect in adults, and this cannot explain the enrichment of Th17 cells in the gut of most people. Recently, Shen et al. reported that the ubiquitous *Alcaligenes faecalis* was sufficient to induce intestinal Th17 cells through the Trim21–Fbxw7 axis^20^. In addition to *Alcaligenes faecalis*, other gut microbial taxa, including *Citrobacter rodentium* and *Escherichia coli* (*E. coli*) O157, potently induce colonic Th17 cell responses in mice through epithelial adherence, as adhesion-defective mutants of these microbes fail to elicit such responses^17^. A separate study found that the generation of intestinal Th17 cells, especially IL-10^+^IFN-γ^–^ Th17 cells, is highly augmented in GF mice following monocolonization with *Propionibacterium* strain P.UF1, and this effect may be attributed to the dihydrolipoamide acetyltransferase (DlaT) expressed in the surface layer of P.UF1 (ref. ^21^). Additionally, bacterial species such as *Clostridium tyrobutyricum*, *Fusobacterium nucleatum*, *Enterococcus gallinarum (E. gallinarum)* and *Bifidobacterium adolescentis* also elicit Th17 cells^22–26^. Notably, this Th17-inducing capacity is not limited to bacteria, as the fungal species *Candida albicans* (*C. albicans)* can also localize close to the colonic epithelium and promote Th17 cell accumulation in monocolonized mice^17^.

Some intestinal bacteria exert precise regulation on Th17 cell differentiation and function through their metabolites as key signalling molecules. Among these, short-chain fatty acids (SCFAs) including acetate, propionate and butyrate have significant effects on both mucosal and systemic immune responses, and their specific roles in modulating Th17 cells have been gradually unveiled^27–29^. Additionally, the secondary BAs generated via microbiota-mediated biotransformation play a key role in regulating Th17 cells. For example, 3-oxolithocholic acid and isolithocholic acid inhibit Th17 cell differentiation by directly interacting with the transcription factor retinoid-related orphan receptor-γt (RORγt)^30,31^, whereas deoxycholic acid (DCA) promotes the differentiation of naive CD4^+^ T cells into pathogenic Th17 cells by increasing the production of endogenous RORγt agonists^32^. Moreover, microbiota-derived tryptophan metabolites, such as indole-3-carboxylate (I3CA) and indoxyl sulfate (I3S), also exhibit the potential to activate Th17 cell responses^33,34^.

#### Th1 cells

The gut microbiota has been shown to be implicated in the orchestration of Th1 responses^10^. *K. pneumoniae* and *K. aeromobilis* elicit gut-specific Th1 cell responses via distinct mechanisms. Specifically, *K. pneumoniae* drives CD11b^−^CD103^+^ DCs-mediated Th1 cell induction via the MyD88-dependent signalling pathway, whereas the induction by *K. aeromobilis* may be associated with flagella and TLR5 (ref. ^35^). Likewise, *E. coli* 35A1 strain isolated from small intestinal mucosa of patients with Crohn’s disease and *Lactobacillus rhamnosus* GG both exhibit potential capacity in Th1 cell induction^36,37^. Additionally, the microbiota-derived metabolites also exert certain regulatory effects on Th1 cells. Acetate and propionate could promote the differentiation of naive CD4^+^ T cells into Th1 cells (characterized by elevated IFNγ and T-bet) in vitro by direct inhibition of histone deacetylase (HDAC) activity. Nevertheless, although acetate treatment enhanced the expression of effector cytokines, these Th1 cells are not highly inflammatory in vivo, and this phenomenon may be related to the concurrent induction of the anti-inflammatory cytokine IL-10 in Foxp3^-^T cells by SCFAs^28^. Notably, a prior review stated that Th1 cells can exhibit a functional shift from an IFNγ^+^IL-10^−^ state towards an IFNγ^+^IL-10^−^ self-regulatory phenotype under appropriate induction, which is consistent with the experimental finding that acetate and propionate drive an expansion of IFNγ^+^IL-10^+^ T cells^28,38^. Concordantly, a series of studies also revealed that butyrate enhances IL-10 production in differentiated Th1 cells. Specifically, butyrate acts on the Th1 cell via G-protein coupled receptor (GPR) 43 and upregulates the transcription factor B lymphocyte-induced maturation protein 1 (Blimp-1), which ultimately leads to IL-10 production^39,40^. Another key metabolite derived from intestinal microbial tryptophan metabolism, indole-3-propionic acid (IPA), directly binds to heat shock protein 70 (HSP70) in Th1 and Th17 cells, impairing mitochondrial oxidative phosphorylation and activating the apoptotic pathway. Consequently, this leads to impaired functionality and reduced numbers of pro-inflammatory Th1 and Th17 cells^41^.

#### Regulatory T cells

Regulatory T (Treg) cells expressing the key transcription factor forkhead box P3 (FOXP3) have a profound impact on intestinal immune homeostasis and tolerance. Specific transcription factors are potentially valuable markers for differentiating the major Treg cell subsets. Thymus-derived Treg (tTreg) cells express nuclear protein Helios and the cell surface protein neuropilin 1 (NRP1), whereas peripherally derived Treg (pTreg) cells typically lack expression of both markers. However, it is worth noting that this distinction is not absolute as the expression of these molecules may fluctuate under the influence of the microenvironment^42^. Both tTreg and pTreg cells respond to the intestinal bacteria, although the tolerance to the microbial community and dietary factors is mainly mediated by pTregs^43^. Among them, RoRγt^+^ pTregs have a stronger response to microorganisms, whereas RoRγt^−^ pTreg mainly maintains tolerance to food antigens. Treg cells expressing GATA3 are mainly positive for Helios, are not tuned by microorganisms, and this population is thought to be a subset of tTregs^42–45^. Previous studies have identified a spectrum of bacterial genera that can promote Treg cells in the colon, such as *Clostridium*, *Bacteroides*, *Bifidobacterium*, and *Helicobacter*^46–49^. More recently, additional bacterial species have been ascribed novel regulatory roles in modulating Treg populations. Liu et.al demonstrated that *Akkermansia muciniphila* (*A. Muciniphila*) alleviates colonic inflammation by upregulating the response of RORγt^+^ Treg cells^50^. Jia et al. reported that *Porphyromonas gingivalis* ingestion remodels the composition of the gut microbiota and downregulates their production of linoleic acid (LA), which inhibits the differentiation of Th17 cells while promoting Treg cell differentiation^51^. Similarly, *Mycobacterium tuberculosis* regulates the production of LA through the *Rv1272c* gene, thereby increasing the function of Treg to suppress the antimycobacterial ability of macrophages^52^. Beyond FOXP3^+^ Tregs, the intestine harbours FOXP3^−^ suppressive T cell populations, type 1 T regulatory (Tr1) cells, which could be modulated by the gut microbiota to exert their gut tissue-reparative capacities^53^. For example, strains such as *Bifidobacterium breve* and *Lactobacillus pentosus* are reported to elicit the generation of IL-10-producing Tr1 cells^54,55^.

In addition to the bacteria cells, microbiota-derived metabolites modulate Treg cells as well. SCFAs are reported to promote the frequency and number of colonic Treg cells and enhance the IL-10-mediated inhibitory function. Mechanistically, SCFAs such as butyrate and propionate inhibit HDAC to enhance histone H3 acetylation at the promoter and conserve non-coding sequence 1 (CNS1) of *Foxp3*. In addition, propionate sensing through GPR43 is another mechanism for Treg cell development^56–58^. Other metabolites, including BAs, polyamines, aromatic amino acids, and some other organic acids, have also been implicated in the regulation of Treg cell function^59^. For instance, 3β-hydroxydeoxycholic acid and isoallolithocholic acid both promote intestinal Treg cell generation^30,60,61^.

#### T follicular helper cells

Tfh cells, which originate from naïve CD4^+^ T cells, can migrate to lymphoid follicles and support B cell activation and differentiation in the germinal centre (GC). In the context of intestinal microenvironment, colonization by intestinal bacterial taxa shapes Tfh cell activation. It has been recognized that SFB, *A. muciniphila* and *Helicobacter hepaticus* could induce Tfh cell differentiation^62–64^. SFB drives the induction of Tfh cells in Peyer’s patches (PPs) in a T cell receptor (TCR)-independent manner. Specifically, SFB limits the IL-2 inhibitory effect on CD4^+^ T cells, thereby enhancing the Tfh cell differentiation process through a DC-mediated pathway^62^. By applying bacteria-specific TCR transgenic models, researchers found that *A. muciniphila* and *Helicobacter hepaticus* are capable of inducing antigen-specific Tfh cells^63,64^. Besides, Fan et al. discovered that commensal SFB induce Th17-to-Tfh17 cell reprogramming within PPs. These Th17-derived Tfh cells exhibit heightened systemic migratory capacity, aberrant GC localization and an enhanced ability to interact with B cells, thereby potentiating autoantibody production^65^. Moreover, the interaction between gut microbiota and Tfh cells is mutual. Microbiota-derived extracellular ATP constrains Tfh cell-dependent helper function by activating the P2X7 receptor expressed on Tfh cells, thereby limiting secretory IgA (sIgA) production. Moderate sIgA secretion is critical for preserving gut microbial diversity and homeostasis. In P2X7-deficient mice, excessive sIgA production disrupts gut microbial composition, ultimately contributing to metabolic dysregulation^66^.

Although the role of metabolites in regulating Tfh cells in the gut–liver axis has been relatively understudied, a recent study provides valuable insights that butyrate supplementation in mice significantly reduces the frequency and number of pathogenic Tfh13 cells within mediastinal lymph nodes in a GPR43-dependent manner^67^. However, the underlying mechanisms governing this interaction remain to be further elucidated.

#### T follicular regulatory cells

T follicular regulatory (Tfr) cells represent a specialized subset of Treg cells that can migrate into GCs to suppress Tfh cell and B cell activities, thereby negatively regulating humoral immunity response. Tfr cells exhibit phenotypic overlap with Tfh cells through the expression of CXC-chemokine receptor 5 (CXCR5), inducible T cell co-stimulator (ICOS), programmed cell death protein 1 (PD-1), and B cell lymphoma 6 (Bcl-6); however, they preserve their regulatory T cell lineage by maintaining Foxp3 expression and other characteristic Treg markers^68^. Several recent studies elucidated that alterations in gut microbiota are implicated in the Tfr/Tfh cell balance^69,70^. In addition, microbiota-derived metabolites play a role in the development of Tfr cells. For instance, Takahashi et al. found that butyrate promotes the differentiation of Treg cells into functionally mature Tfr cells through inhibition of HDAC activity and augmentation of histone acetylation at key Tfr cell genes such as *Bcl6*, *Cxcr5* and *Tcf7* (ref. ^71^).

#### Other Th cells

Several other Th cell subsets, such as Th2, Th9 and Th22 cells, have also been demonstrated to be regulated by the intestinal microbiota. For instance, the administration of *Clostridium butyricum Miyairi588* or *B. adolescentis* induced protective Th2 cell responses that alleviate intestinal inflammation in the dextran sulfate sodium (DSS)-induced colitis model^48,72^. Th9 cells that secrete IL-9 are the key effector cells of succinate-mediated colonic inflammation. Specifically, gut microbiota-derived succinate accumulates in the colon owing to an imbalance between succinate-producing bacteria and succinate-metabolizing bacteria in ulcerative colitis (UC). This elevated succinate activates the succinate receptor SUCNR1 on CD4^+^ T cells, triggering the differentiation of naive CD4^+^ T cells into colitogenic Th9 cells, as this pathogenic process can be alleviated by SUCNR1 antagonists and anti-IL-9 antibodies^73^. Besides, IL-22-secreting Th22 cells can be induced by bactericidal/permeability-increasing protein (BPI)-mediated DC activation, an effect that may be potentiated by increased diversity of intestinal microbiota^74^.

### CD8^+^ T cells

Cytotoxic CD8^+^ T cells are critical mediators of immune surveillance against intracellular pathogens and malignant cells^75^. The unique intestinal microenvironment shapes the distinct differentiation capacity and cytotoxicity of CD8^+^ T cells. For instance, a commensal consortium comprising 11 healthy individual-derived bacterial strains has been shown to induce IFNγ-producing CD8^+^ T cells in the intestinal tract^76^. However, gut dysbiosis may disrupt this process. A study by Yu et al. revealed that microbial dysbiosis overstimulates CD8^+^IFNγ^+^ T cells, thereby triggering chronic inflammation, early T cell exhaustion and eventually accelerated tumorigenesis^77^. Notably, a crucial probiotic *A. muciniphila* plays a significant role in regulating intestinal CD8^+^ T cells during diseases. A study has verified that either pasteurized *A. muciniphila* or its specific outer membrane protein Amuc_1100 mitigates the inflammatory infiltration of CD8^+^ T cells in the colon to alleviate colitis. In the tumour microenvironment of colitis-associated colorectal cancer (CAC), they significantly increased the proportion of CD8^+^ T cells in the MLN while inducing the secretion of TNF and the downregulation of PD-1 to exert cytotoxic effect^78^.

Microbiota-derived metabolites also correlate with CD8^+^ T cell response. Mevalonate and dimethylglycine derived from aforementioned 11 bacterial strains may serve as key bridges to mediating the functional activation of CD8^+^IFNγ^+^ T cells^76^. Besides, SCFAs are emerging as critical regulators of CD8^+^ T cell function. Exogenous acetate supplementation enhances oxidative phosphorylation, glycolysis and secretion of IFNγ and granzyme B (GzmB) in virus-specific CD8^+^ T cells, as evidenced in Abx and GF mice^79^. Butyrate has similarly been implicated in potentiating CD8^+^ T cell immunity, with its effects mediated through multifaceted mechanisms, including the promotion of memory cells, enhancement of cytotoxicity and protection from T cell exhaustion^80–83^. Tryptophan-derived metabolites indole-3-lactic acid (ILA) and IPA further expand this regulatory network by modulating CD8^+^ T cell function via epigenetic modifications^84,85^. Specifically, ILA increases trimethylated H3K27 enrichment at the regulatory regions of *Saa3* to inhibit its expression, which regulates cholesterol metabolism, thereby enhancing CD8^+^ T cell effector functions^84^. IPA enhances CD8^+^ T cell stemness and anti-tumour activity by increasing H3K27 acetylation at the *Tcf7* super-enhancer^85^. In contrast, DCA, a secondary bile acid modified by intestinal microbiota such as *Clostridium scindens*, suppresses CD8^+^ T cell effector functions by potentiating plasma membrane Ca^2+^ ATPase (PMCA) to impair Ca^2+^-NFAT2 signalling, thereby promoting colorectal cancer growth^86^. Of particular relevance to CLD, gut-activated CD8^+^ T cells have been shown to migrate from the intestine to the liver via the enterohepatic circulation — a one-way pathway that enables these cells to participate in hepatic immune responses^87^.

### Innate-like lymphocytes

#### Natural killer T cells

Natural killer T (NKT) cells are CD1d-restricted and lipid antigen-reactive lymphocytes that express TCR as well as NK cell-associated markers^88^. These cells are mainly categorized into two subsets: type I NKT cells, also referred to as invariant NKT (iNKT) cells, and type II NKT cells. Compared with other immune organs, the liver harbours a remarkably high frequency of NKT cells^89^. The hepatic NKT cells can be influenced by gut microbiota through multiple mechanisms. Ma et al. found that gut microbe-mediated bile acid metabolism regulates CXCL16 expression in liver sinusoidal endothelial cells (LSECs), thereby influencing hepatic accumulation of CXCR6^+^ NKT cells, which can generate IFNγ to exert antitumour effects^90^. Similarly, the administration of *A. muciniphila* inhibits non-alcoholic steatohepatitis-associated tumorigenesis, with this protective effect mediated by CXCR6^+^ NKT cells^91^. In addition, the presentation of glycosphingolipids derived from intrahepatic Bacteroidetes to hepatic NKT cells activates CCL5 signalling, which forms a “microbiota–glycosphingolipid–NKT–CCL5” axis. This cascade recruits additional immune cells and serves as the foundation for hepatic immune homeostasis^92^. Beyond the liver, NKT cells are also present in the intestine, where they interact with the gut microbiota through a two-way communication process^93^. Abnormal accumulation of iNKT cells was observed in the colonic LP of GF mice, revealing the impact of microbial exposure on NKT function^94^. In turn, several studies have announced the alternation of commensal microbiota in NKT cell-deficient mice^95–97^. However, a recent study yielded conflicting results that Vα14 Tg (with an increasing number of iNKT cells) and *CD1d*^*−/−*^ mice showed no significant changes in intestinal microbiota composition compared with their littermate controls^98^. These discrepancies might be the application of littermates and cross-fosterage, and the authors further suggested that maternal transmission and caging conditions may impact the microbiota composition more than the genotype.

#### MAIT cells

MAIT cells are a subset of T cells that express a semi-invariant TCR. MAIT cells can be activated either by recognizing the microbial metabolites of vitamins B2 (riboflavin) through major histocompatibility complex class I-related molecule (MR1) presentation, or cytokines such as IL-12 and IL-18, in a TCR-independent manner^99^. They are widely distributed in human blood, liver and the intestinal LP. MAIT cells are significantly reduced in GF mice compared with SPF mice, suggesting their dependency on microbiota^100,101^. Indeed, the development of MAIT cells relies on early-life exposure to riboflavin-synthesizing commensals, and it is proposed that the production of microbial riboflavin derivative 5-(2-oxopropylideneamino)-6-D-ribitylaminouracil (5-OP-RU) is a determining factor. Even if GF mice are exposed to the microbiota during adulthood, the development of MAIT cells remains permanently impaired^102,103^. The composition of intestinal bacterial community alterations, particularly shifts in riboflavin-producing bacterial populations, leads to differences in the distribution and functionality of MAIT cells^104,105^. For example, the luminal riboflavin-producing bacteria proliferation in mice with DSS-induced colitis leads to elevated levels of 5-OP-RU, which can rapidly cross the intestinal barrier and promote MAIT cell activation^106^.

Beyond the intestine, the liver is another key tissue where MAIT cells exert important functions, with their activation and regulation closely linked to microbial metabolites and local cellular interactions. In the liver, MAIT cells are distributed throughout the entire parenchyma, where they can be activated by primary hepatocytes, hepatic stellate cells, hepatic sinusoidal endothelial cells and bile-duct epithelial cells through MR1-dependent mechanisms. Among these cells, hepatocytes appear to be the most efficient antigen-presenting cells for MAIT cells. It is revealed that hepatocytes possess the capacity to convert the bacterial metabolic precursor 5-amino-6-ribitylaminouracil (5-A-RU) into the active antigen 5-OP-RU, the most potent stimulatory antigen for MAIT cells^107^. Thus, gut microbial dysbiosis may enhance the delivery or local generation of MAIT cell-activating metabolites in the liver, thereby triggering aberrant MAIT cell activation. Such dysregulated activation has been implicated in the pathogenesis of immune-mediated liver diseases.

#### γδ T cells

γδ T cells, defined by their TCR consisting of γ and δ chains, do not require classical MHC molecules for antigen perception and exhibit broad antigen recognition capacity. The γδ T cells account for 1–5% of T cells in human peripheral blood, but are abundant in the liver and intestine^108,109^. In the context of intestinal immunity, accumulating evidence indicates that γδ T cells respond to pathogenic bacteria invading the intestine by producing IL-17 (refs. ^110,111^). Importantly, the production of IL-17 can be modulated by microbial metabolites. Propionate directly represses IL-17 production by mouse intestinal γδ T cells through an HDAC-dependent mechanism, and also reduces IL-17 production by human peripheral γδ T cells from IBD patients^112^. Beyond effector responses, the role of γδ T cells in maintaining intestinal microbiota homeostasis is supported by observations in γδ T cell-deficient (γδ^−/−^) mice, where intestinal microbiota composition is altered, and this dysbiosis in turn leads to immune dysregulation and impaired small intestinal tolerance^113^. Notably, the interaction between γδ T cells and the microbiota is highly complex. The relationship is not solely determined by the anatomical location of γδ T cell subsets or their primary effector functions. It is further intertwined with the developmental lineage of γδ T cells, tissue-specific microenvironmental cues, and dynamic bidirectional regulatory loops that vary across physiological and pathological contexts^114^. Moreover, the critical role of γδ T cells in the gut–liver axis has been increasingly elucidated in recent years. A series of studies have demonstrated that alterations in gut bacterial load modulate the frequency of IL-17-producing γδ T cells in the liver, thereby influencing the progression of liver disease^115–117^.

## T cell plasticity

T cell subsets are typically defined by their expression of lineage-specific transcription factors and effector molecules. However, these phenotypic and functional attributes are not stable but rather flexible. Specific environmental cues enable shifts in the identity of T cells, leading to the acquisition of other features from T cell subsets, which is known as T cell plasticity^118^. In the intestine, such plasticity is particularly prominent among CD4^+^ T cell subsets. For instance, researchers have identified Th17/Th1 and Th17/Tfh hybrid cell populations, as well as Treg cells that originate from Th17 cell precursors^119^. This plasticity is regulated by factors including the gut microbiota, dietary metabolites, cytokines and epigenetic modifications. Among these, commensal microbes are recognized as pivotal regulators of this process^119,120^. However, it remains unclear whether the microbiota acts as a necessary trigger for T cell subset conversion or simply as one of multiple contributing factors. Studies such as that by Lui et al. demonstrate that the conversion of CD8^+^ to CD4^+^ T cells can initiate in mesenteric lymph nodes (MLNs) in the absence of microbiota. Although the initial conversion is microbiota independent, the subsequent homing and expansion of these cells within the intestinal environment critically rely on microbial signals^121^. Thus, further studies are warranted to elucidate the precise mechanisms and identify the specific microbial taxa involved.

## Role of intestinal microecology in chronic liver disease

**Fig. 2:**
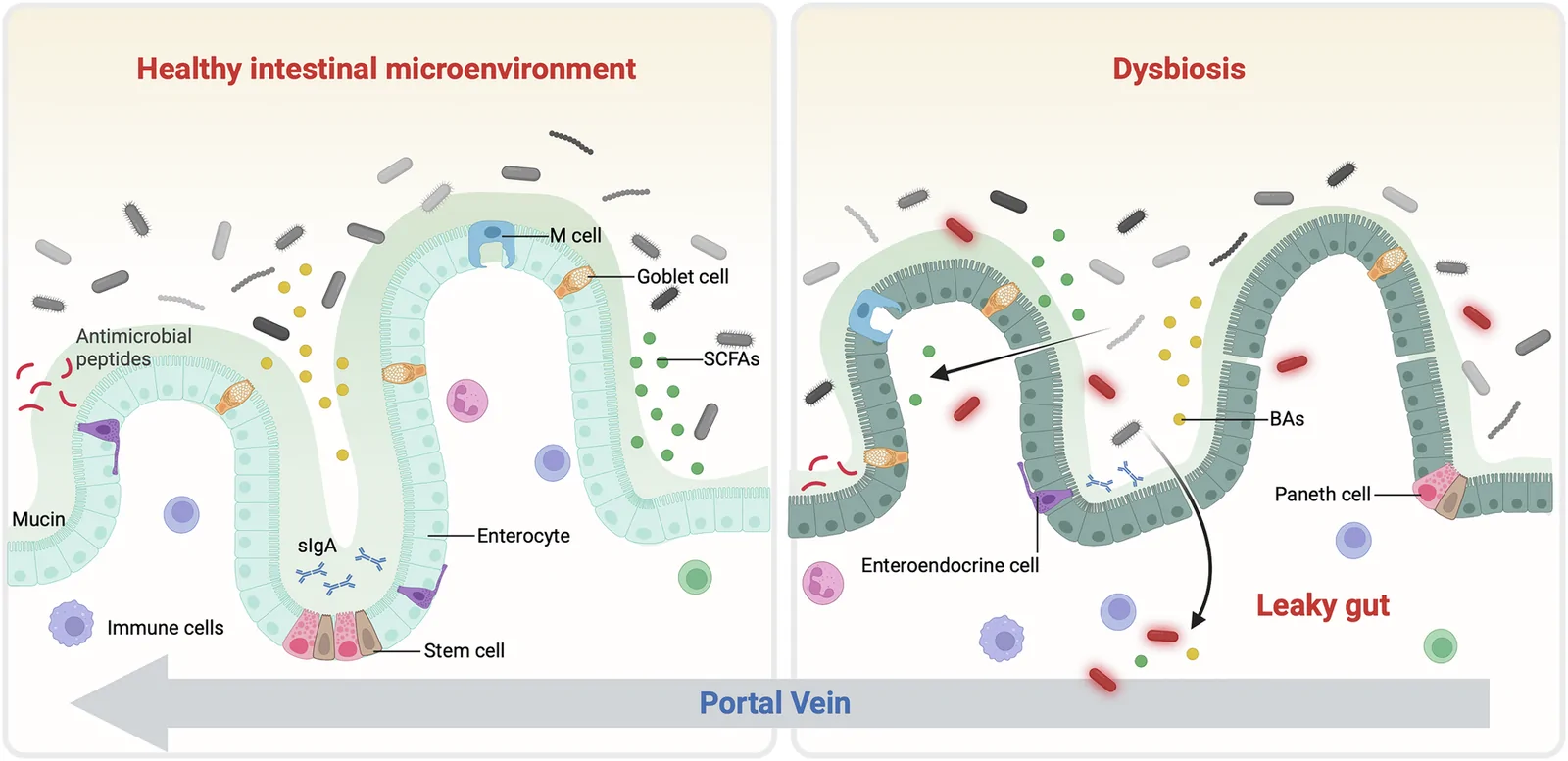
Intestinal barrier provides defence to the microbial translocation. The intestinal barrier integrity is maintained by physical, chemical, immunological, and microbial barriers. Intestinal epithelial cells, together with intercellular junctions, constitute the physical barrier. The chemical barrier mainly consists of the antimicrobial peptides by Paneth cells, mucins and secretory immunoglobulin A (sIgA). The immune barrier is formed by gut-associated lymphoid tissues and immune cells distributed throughout the intestine, and a healthy and balanced gut microbiota constitutes the biological barrier. Under circumstances of inflammation or immune imbalance, gut dysbiosis often coexists with intestinal barrier dysfunction. The microbiota and their metabolites may cross the disrupted intestinal barrier and enter into the portal vein, then translocate to systemic organs and cause diseases. BA, bile acid; SCFA, short-chain fatty acid.

As an interface between the internal milieu and the external environment, the intestine has to process dietary antigens while maintaining symbiosis with a vast community of microorganisms. The community, encompassing bacteria, fungi, archaea, viruses and protozoa, collectively constitutes the intestinal microbiota, a core component of the gut microecosystem^122,123^. Within the host, the microbiota fulfils essential physiological functions, including nutrient metabolism, intestinal defence modulation and immune regulation, thereby exerting profound effects on host health. To maintain host–microbiome homeostasis, the intestinal barrier serves as a multifaceted defence system integrating physical, chemical, immunological and microbial barriers^124^ (Fig. 2). The mucus layer — primarily composed of mucins secreted by goblet cells — forms as the first line of protection against pathogen invasion^125^. Beneath this layer, intestinal epithelial cells (IECs) including absorptive enterocytes, goblet cells, Paneth cells, enteroendocrine cells and microfold cells (M cells) constitute the structural foundation of the barrier. Together with intercellular junctions including tight junctions, adherens junctions and desmosomes in the intestinal epithelial layer, the cells form a physical barrier with selective permeability^126^. The chemical barrier mainly consists of mucins, secretory immunoglobulin A (sIgA), and the antimicrobial peptides such as defensins and lysozyme produced by Paneth cells^127^. The immune barrier is formed by gut-associated lymphoid tissues and immune cells distributed throughout the intestinal tract. Finally, a healthy and balanced gut microbiota functions as a key component of the host’s biological defence system^124^.

Growing evidence supports the critical role of disrupted intestinal barrier integrity, commonly referred to as “leaky gut”, in the pathogenesis of multiple liver disease entities^128,129^. The barrier impairment allows bacteria, food particles and toxins to translocate into the systemic circulation^130^. Microbiota-derived antigens serve as an important mediator to drive the progression of hepatic immune pathology through a molecular mimicry mechanism. For instance, evidence from Terjung et al. indicates that immune responses in PSC and autoimmune hepatitis (AIH) may arise from cross-reactivity between microbial antigens and host components^131^. Similarly, the pyruvate dehydrogenase complex E2 (PDC-E2) of *E. coli* exhibits cross-reactivity with the human counterpart at the TCR molecular level to trigger primary biliary cholangitis (PBC) progression^132^. Besides, bacteria-produced toxins in the systemic circulation may also be a contributing factor to certain extraintestinal diseases. For example, barrier disruption increases the entry of endotoxins from intestinal Gram-negative bacteria into the bloodstream, resulting in persistent chronic inflammation and progressive liver injury^133^.

**Fig. 3:**
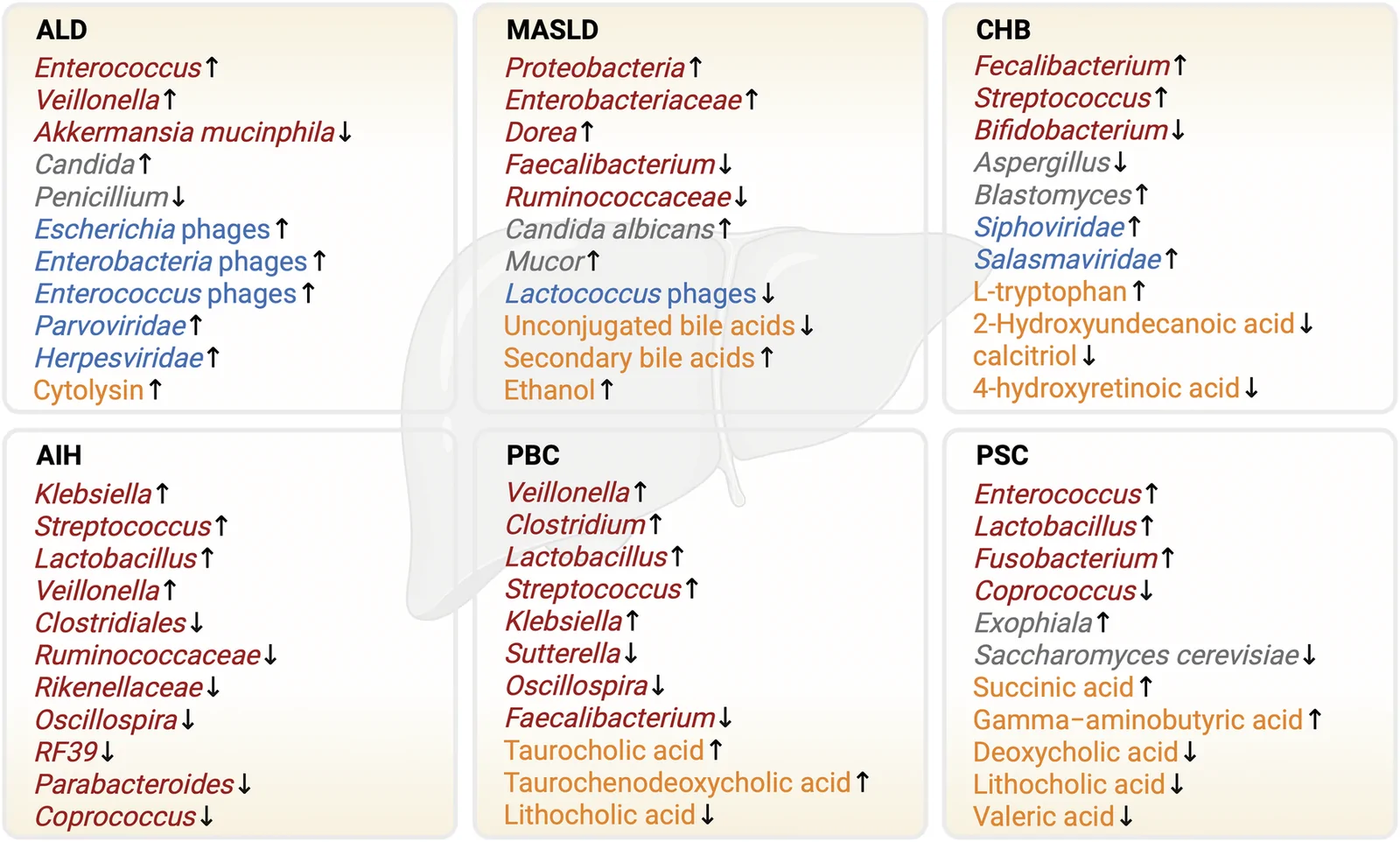
Alternation of gut microbiomes in different chronic liver diseases. Red represents bacteria; grey represents fungi; blue represents virus; yellow represents metabolites. AIH, autoimmune hepatitis; ALD, alcohol-associated liver disease; CHB, chronic hepatitis B; MASLD, metabolic dysfunction-associated steatotic liver disease; PBC, primary biliary cholangitis; PSC, primary sclerosing cholangitis.

Gut dysbiosis, defined as imbalances in the microbial ecology, frequently coexists with intestinal-barrier dysfunction^134^. Numerous studies have reported alterations in gut microbiota composition in CLD (Fig. 3), characterized by three key features: reduced microbial diversity, overgrowth of pathogenic bacteria and depletion of commensal taxa^135^. For example, there was a reduction in *Faecalibacterium* and *Bacteroides*, and an enrichment of *Klebsiella* and *Veillonella* accompanied by a significant diminution of within-individual microbial diversity in PBC patients^136^. Likewise, the decrease in *Faecalibacterium* abundance was also observed in both ALD and non-alcoholic fatty liver disease (NAFLD), and it is notable that the abundance of *C. albicans* increased^137–139^. In CLD, changes in gut microbiota are not limited to composition but also involve its function, including metabolic outputs (e.g. production of SCFAs, bile acids and aromatic amino acids) and the expression of certain genes conferring antibiotic resistance and virulence^140–142^. As one of the most extensively investigated metabolites, SCFAs display disease-specific profile shifts in NAFLD, ALD and AILD, along with consequential impacts on the host’s immunity^143–145^. On the functional front of microbial virulence and antibiotic resistance, Lee et al. demonstrated that advanced cirrhosis drives enrichment of pathogenic enterotypes with elevated virulence factors (e.g. ascorbate degradation, LPS biosynthesis) and microbiome resistomes with antimicrobial resistance genes (e.g. resistance against aminoglycoside, fluoroquinolone, macrolide and nitroimidazole)^146^. Importantly, gut–liver crosstalk is not unidirectional: the intestine influences hepatic physiology, whereas the liver communicates with the gut via bile flow and the release of circulating metabolites. However, there is still debate over whether leak gut or dysbiosis is a cause or an effect of liver diseases^134^.

## Gut microbiota–T cell interactions in chronic liver disease

Given the increasing number of studies linking microbial cues to T cell-mediated immune alterations in chronic liver disease, the reported interactions have become highly diverse across disease contexts, microbial sources and T cell subsets (Table 1). In the following sections, we discuss these microbiota–T cell axes according to major chronic liver disease categories, with emphasis on their disease-specific immune consequences.

**Table 1:** Microbial signals regulate T-cell immunity in chronic liver diseases.

Liver disease	Microbial cues	Immunological effect	Proposed pathological or protective role	Refs.
AIH	*Enterococcus gallinarum*	Inducing Th17 response and autoantibody production	Leading to autoimmune pathology related to AIH	^25,152^
	*Lactobacillus reuteri-*derived I3A	Triggering IFNγ^+^ CD8^+^ T cell activation	Promoting AIH-like pathology in *Tet2*^*ΔVAV*^ mice	^153^
	ICA	Restricting of effector T cells	Ameliorating AIH	
PBC	FMT from PBC patients	Abundant infiltration of CD4^+^ T cells, CD11b^+^ granulocytes and CD56^+^ NK cells around the bile ducts	Eliciting bile duct injury	^158^
	*Escherichia coli* or *Novosphingobium aromaticivorans* protein lysates	Stimulating circulating CD4^+^CXCR5^+^ Tfh cells from PBC patients in vitro	Potentially promoting autoimmune reactions	^159^
PSC	Heat-killed *Enterococcus faecalis*, *Staphylococcus aureus* or *Candida albicans*	Elevating the proportion of Th17 cells in PBMC from PSC	Promoting Th17-immune activation	^161^
	*Klebsiella pneumoniae*, *Proteus mirabilis* and *Enterococcus gallinarum*	Inducing hepatic Th17 priming	Promoting hepatobiliary inflammation	^9^
	*Lactobacillus gasseri*	Inducing IL-17A production by intrahepatic γδ T cells	Promoting hepatic inflammation and fibrosis	^116^
ALD	Faecal bacterial antigens, toxins and metabolites from patients with ALD	Peripheral MAIT‑cell depletion and hyperactivation	Compromising antibacterial immunity	^168^
	*Candida albicans*	Migration of intestinal *Candida albicans*-specific Th17 cells to the liver and activation of Kupffer cells via IL-17/IL-17RA signalling	Exacerbating ethanol-induced liver injury	^169^
MASLD	Gut microbiota-derived lipid antigens	Activating hepatic γδT-17 cells by hepatocytes via CD1d	Accelerating MASLD	^115^
	Faecal BEs from MASLD-HCC patients	Expanding Treg cells and attenuating CD8^+^ T cells in vitro	Promoting a peripheral immunosuppressive phenotype that may impair antitumour immunity	^179^
CHB	BEs from CHB patients	BEs from non-cirrhotic patients: expanding of Th17 cells in vitro; BEs from cirrhotic patients: attenuating Th1 expansion in vitro	Disturbing peripheral T cell homeostasis and contributing to disease progression	^184^
	Probiotics (*B. longum*, *E. faecalis* and *Lactobacillus acidophilus*)-derived spermidine	Provoking intrahepatic IFNγ^+^CD4^+^ T cell immune response	Promoting HBV clearance and inhibiting HBV replication	^185^

AIH, autoimmune hepatitis; ALD, alcohol-associated liver disease; BEs, bacterial extracts; CHB, chronic hepatitis B; FMT, faecal microbiota transplantation; HBV, hepatitis B virus; HCC, hepatocellular carcinoma; I3A, indole-3-aldehyde; ICA, indole-3-carboxaldehyde; MASLD, metabolic dysfunction-associated steatotic liver disease; PBC, primary biliary cholangitis; PSC, primary sclerosing cholangitis.

### Autoimmune liver diseases

AILDs comprise a group of chronic inflammatory hepatobiliary disorders mediated by dysregulated autoimmune response. According to their distinct histopathological features, imaging findings and clinical manifestations, AILDs are classified into AIH, PBC, PSC and IgG4-related cholangitis. Although relatively uncommon, the prevalence of AILD is rising in certain regions, imposing an increased clinical burden^147^. In the past years, the critical role of the gut microbial dysbiosis in AILD has been well documented^148^. However, the interplay between the gut microbiota and T cells in this context has not yet been systematically reviewed.

AIH is characterized by immune-mediated hepatocellular injury, predominantly driven by autoreactive T cell responses, leading to hepatocyte necrosis and hepatic fibrosis^149,150^. Our prior research identified alterations in the composition and function of the intestinal microbiota in Chinese patients with AIH, and suggested that bacteria may have immunomodulatory functions^151^. In experimental models, Manfred Vieira et al. demonstrated that the gut pathobiont *E. gallinarum* translocated to the liver and systemic tissues, and this translocation was associated with enhanced Th17 responses and systemic autoantibody production. Importantly, *E. gallinarum* DNA was also detected in the livers of AIH patients, and co-culture of *E. gallinarum* with human hepatocytes induced various autoimmune-promoting factors that recapitulated the findings observed in mice^152^. Furthermore, specific antibodies against *E. gallinarum* were also produced in the serum of AIH patients^25^. Pandey et al. also discovered that *Lactobacillus reuteri* (*L. reuteri*) translocated into the liver, where its metabolite indole-3-aldehyde (I3A) may trigger the IFNγ-producing CD8^+^ T cell to induce AIH-like pathology in *Tet2*^*ΔVAV*^ (haematopoietic *Tet2* deficiency) mice, a model that resembles AIH^153^. Recently, another microbial metabolite, ICA, has been shown to improve AIH by constraining effector T cells through targeted stimulating aryl hydrocarbon receptor (AhR) in T lymphocytes^8^. It is worth noting that T cells also have an impact on the microbiota in AIH. Centa et al. reported that mice lacking medullary thymic epithelial cells (*Traf6*^*ΔTEC*^) exhibited impaired central tolerance and generated autoreactive T cells. These aberrant thymic T cell compartment remodels gut microbiota, which increases the susceptibility to AIH. Furthermore, microbiota-mediated hepatic Foxp3^+^ T cells also correlate with the severity of AIH^154^. These findings collectively establish a bidirectional interaction between the gut microbiota and T cells in AIH pathogenesis.

During the pathogenesis of PBC, T cells play a crucial role as the core immune cells that mediate damage to the bile duct epithelial cells and maintain chronic inflammation^155^. Concurrently, significant perturbations in the gut microbial community have also been implicated in the pathogenic process of PBC^136,156,157^. A recent study found that mice that received faecal microbiota transplantation (FMT) from PBC patients exhibited similar bile duct responses to 2-octynoic acid-bovine serum albumin (2OA-BSA)-induced PBC mouse model, and both groups showed an obvious CD4^+^ T cell infiltration around the bile ducts compared with mice that received FMT^158^. Despite a lack of direct evidence, these findings suggest that the gut microbiota derived from PBC patients is capable of eliciting T cell-mediated immune responses within the liver. Zhou et al. demonstrated that the heat-killed whole cells or lysates of the bacteria associated with PBC initiation, such as *E. coli* and *Novosphingobium aromaticivorans*, contribute to the aberrant activation of circulating CD4^+^CXCR5^+^ Tfh cells of PBC patients in vitro^159^.

PSC is a chronic inflammatory cholestatic liver disease that often coexists with ulcerative colitis (PSC/UC). Patients with PSC exhibit decreased faecal microbial α-diversity, accompanied by shifts in metabolic profiles^160^. Specific pathogenic microorganisms, including *Staphylococcus, Streptococcus* and *C. albicans*, are detected in bile and portal tracts of patients with PSC. Notably, in vitro studies have demonstrated that stimulation with heat-killed *C. albicans* significantly elevated the proportion of Th17 cells in peripheral blood mononuclear cells (PBMCs) from patients with PSC compared with healthy controls and patients with PBC^161^. Apart from biliary pathogens, *Klebsiella pneumoniae*, *Proteus mirabilis* and *Enterococcus gallinarum* are prevalently present in faeces from patients with PSC. Among these, *Klebsiella pneumoniae* possesses strain-specific epithelial pore-forming capacity, enabling bacterial translocation. Specifically, when a mixture of these three bacteria was inoculated into GF mice, epithelial barrier disruption facilitated the influx of microbial components and by-products into the liver, thus may contribute to a robust Th17 cell response in the mouse liver. However, bacteria were not detected in the liver by bacterial culturing, polymerase chain reaction (PCR), or fluorescence in situ hybridization (FISH), possibly because of the prevention by the gut–vascular barrier^9^. In another study applying *Mdr2*^*−/−*^ mice, *Lactobacillus gasseri* (*L. gasseri*) was enriched in liver tissues relative to control mice, which may be attributed to the increased gut permeability and subsequent bacterial translocation from the intestine lumen to the liver. The translocated *L. gasseri* induces the production of IL-17A in γδ T cells to promote inflammation and fibrosis, and this pathogenic effect can be attenuated by the neutralizing antibodies against γδ TCR^116^. Collectively, the microbial dysbiosis in AILD may either act as initiating factors for T cell dysfunction or represent consequences of aberrant T cell activity. Although research in this field remains in its early stages, the gut–liver–immune axis holds promise as a therapeutic target for AILD.

### Alcohol-associated liver disease

The high prevalence of alcohol consumption renders ALD a major public health concern. Approximately 3.5% of the global population suffer from ALD, with a 5-year mortality rate exceeding 50%. ALD is a progressive process, ranging from reversible stages of alcoholic fatty liver to alcoholic hepatitis and alcoholic cirrhosis, which may eventually culminate in HCC^162^. Owing to the addictive properties of alcohol and the considerable difficulties in ALD treatment, research efforts have been increasingly focused on the intestinal microbiota as a novel therapeutic target^163^. A review emphasized that the complex interplay between the intestinal immune system and microbiota plays a pivotal role in ALD^164^. This section specifically explores the crosstalk between T cells and the intestinal microbiota in the context of ALD.

Alcoholism causes the loss of duodenal CD8^+^ T cells and compromises intestinal barrier function^165–167^. The intestinal leakage facilitates the translocation of intestinal bacteria, microbial antigens and metabolites into the systemic circulation in ALD patients. This increasing systemic exposure to the bacterial products significantly heightens the contact frequency with MAIT cells, which induces the depletion and hyperactivation of peripheral blood MAIT cells, leading to the compromised anti-infective capacity. However, the number of intrahepatic MAIT cells is preserved with the preference of portal accumulation, which is considered to be protection from bacterial translocation^168^. Beyond bacteria, dysbiosis of fungal communities also contributes to ALD pathogenesis. Zeng et al. discovered that ethanol intake promotes intestinal fungal dysbiosis and induces the generation of *C. albicans*-specific Th17 cells in the intestine. These Th17 cells migrate to the liver, where they act on hepatic Kupffer cells through the IL-17/IL-17RA signalling pathway, resulting in exacerbated liver damage^169^. Despite gaps in mechanistic understanding, probiotic supplementation emerges as a promising strategy to mitigate ALD, underscoring the clinical relevance of targeting gut microbiota–T cell crosstalk.

### Metabolic dysfunction-associated steatotic liver disease

The term MASLD was proposed in 2023 to replace NAFLD, a change that underscores the critical role of underlying metabolic dysfunction in the disease pathogenesis^170^. New evidence indicates a high consistency between the definitions of NAFLD and MASLD — more than 99% of patients with NAFLD meet the criteria for MASLD^171^. Therefore, we apply MASLD instead of NAFLD in the following content. MASLD encompasses a spectrum of hepatic disorders, ranging from simple steatosis, metabolic dysfunction-associated steatohepatitis, with potential progression from fibrosis to cirrhosis, and ultimately HCC^172^. Strikingly, approximately 38% of the global adult population suffers from MASLD, with the burden of related complications rising worldwide^173,174^. Recent studies have demonstrated that MASLD is a multifactorial disease driven by the interaction of metabolic dysfunction, gut microbiota perturbations and immune system abnormalities^175^. Mice fed a high-fat diet or a methionine choline-deficient diet show decreased Th17 cell counts in small intestinal lamina propria. Th17 cells are recognized as protective factors in intestinal barrier integrity, and their depletion promotes the microbial migration that may accelerate MASLD progression^176,177^. γδT-17 cells are another key source of IL-17 in the liver, and their frequencies are tightly regulated by the gut microbiota. Lipid antigens derived from intestinal microbiota are presented to the hepatic γδT-17 cells by hepatocytes via CD1d, and the stimulated γδT-17 cells then contribute to accelerating the development of MASLD in high-fat diet or high-fat/high-carbohydrate diet mouse models^115^. The progression of MASLD is associated with gut microbial dysbiosis and corresponding alterations in peripheral immune status^178^. For instance, a significant negative correlation was found between blood resting Tregs and faecal propionate or acetate levels in patients with metabolic dysfunction-associated steatohepatitis^143^. Furthermore, Behary et al. reported that the faecal bacterial extracts of patients with MASLD-HCC induce immunosuppressive phenotypes ex vivo, including the expansion of Treg cells and the attenuation of CD8^+^ T cells, which are tightly linked to the SCFAs^179^.

### Viral hepatitis

Hepatitis B virus (HBV) constitutes one of the foremost aetiological agents of chronic viral hepatitis. Epidemiological evidence indicates that chronic hepatitis B (CHB) affects more than 250 million people worldwide, and individuals with chronic HBV infection carry a 5- to 100-fold risk of developing HCC^180,181^. Both gut microbiota and T cell immunity are disturbed in the context of HBV infection. Patients with CHB exhibited gut dysbiosis, typified by an enrichment of pro-inflammatory taxa and a depletion of beneficial bacteria^182^. Clinically, these patients manifest impaired HBV-specific T cell responses and general T cell dysfunction^183^. Mounting evidence underscores a functional interplay between these two pathological hallmarks in CHB^182,184^. Shen et al. proposed that gut microbiota affects the balance of peripheral T cell subsets, which may exacerbate hepatic fibrosis and disease progression. Specifically, bacterial extracts from patients with non-cirrhotic CHB significantly promoted the expansion of Th17 cells, whereas bacterial extracts from patients with cirrhotic CHB inhibited Th1 cells^184^. It is worth noting that targeting the interaction between these two compartments has already demonstrated therapeutic potential. Wang et al. discovered that a probiotic consortium comprising *B. longum*, *E. faecalis* and *Lactobacillus acidophilus* and its metabolite spermidine effectively suppresses HBV replication by augmenting IFNγ^+^CD4^+^ T cell immunity^185^. Although the correlation between T cells and the gut microbiota in CHB remains incompletely characterized, further explorations will propel the clinical translation.

## Therapeutic targets and future perspectives

### Interventions targeting microbiota

Microbial modulators represent promising adjunctive therapeutic strategies for CLD by targeting the gut–liver axis. A systematic review reported that probiotics, prebiotics and synbiotics alleviate pathological features of MASLD through diverse mechanisms^186^. Probiotics are live microorganisms, and they are beneficial to the host’s organ health in a suitable dose^187^. A recent study by Wu et al. demonstrated that the supplementation of *L. acidophilus* not only attenuated the bile duct ligation-induced cholestatic liver damage in mice but also improved the liver function of patients with cholestasis^188^. Besides, daily oral *L. rhamnosus* GG is reported to alleviate liver injury in moderate alcohol-associated hepatitis^189^. The definition of prebiotics was updated in 2017 as a substrate that is selectively utilized by host micro-organisms conferring health benefit, including fructo-oligosaccharides, inulin and galacto-oligosaccharides^190^. A randomized crossover trial revealed that Amazonian berry camu-camu significantly reduces hepatic fat accumulation in individuals with overweight and hypertriglyceridaemia and decreases the level of AST and ALT, and this effect is related to the changes in the composition and function of gut microbiota, suggesting that camu-camu has a prebiotic-like effect^191^. Moreover, inulin improves liver enzymes in patients with obesity who engage in physical exercise and significantly regulates *Bifidobacterium*, *Dialister* and *Catenibacterium* genera^192^. However, the improvement of liver damage did not occur in early ALD, although inulin leads to specific alterations in the gut microbiota^193^. Finally, synbiotics refer to the combination of probiotics and prebiotics. A study suggested that synbiotic supplementation augmented the efficacy of lifestyle intervention in MASLD patients^194^, although Scorletti et al. demonstrated that fructo-oligosaccharides plus *Bifidobacterium* did not reverse hepatic fat accumulation or fibrosis^195^.

FMT is increasingly being investigated as a safe intervention to optimize the gut microbiota composition and function, with the potential to regulate CLD development through the gut–liver axis^196^. Nevertheless, several studies indicated that the FMT from healthy donors failed to improve insulin resistance and hepatic proton density fat fraction in patients with MASLD, but it is effective in restoring intestinal permeability^197,198^. In another trial that enrolled individuals with PSC, the patients showed increased bacterial diversity and decreased ALP levels after receiving FMT intervention^199^. Moreover, FMT also seems to serve as a protective measure for hepatic encephalopathy^200,201^.

### Interventions targeting T cells

Aberrant T cell responses represent a key driving factor for intrahepatic inflammation and fibrosis. A recent study identified an expanded population of pathogenic PD-1^+^CD8^+^ T cells in the liver that promotes AILD progression by triggering hepatocyte pyroptosis. Eliminating these cells via chimeric antigen receptor (CAR)-T therapy effectively ameliorated disease severity in experimental models^202^. Besides, expanding Treg cells to suppress overactivated immunity emerges as a promising therapeutic strategy for AILD as well. A clinical case report documented the infusion of autologous Treg cells into a 48-year-old female patient diagnosed with refractory PSC/UC — a patient who had previously failed to respond to multiple immunosuppressants and biologics. Surprisingly, the clinical results showed a significant reduction in serum liver enzyme levels at 2–4 weeks post-infusion^203^. Additionally, low-dose IL-2 therapy that selectively supports the expansion and function of Tregs also holds potential, and this has already achieved initial success in both basic research and clinical treatment^204–206^.

### Combined microbiota and T cell-targeted therapies

Gut microbiota modulation and T cell-targeted therapies have attracted considerable clinical attention, each showing great potential in the pathological progression of CLD. Notably, integrating gut microbiota modulation and T cell therapy, this synergy may enhance efficacy, reduce adverse effects and address the multifactorial nature of CLD, holding significant promise for advancing CLD treatment. Admittedly, our current understanding of the interaction between the gut microbiota and T cells in CLD remains incomplete. The network involving the gut microbiota, T cells and the liver is highly complex and bidirectional. Consequently, targeting a single component may initiate an unanticipated cascade of effects. For instance, the therapeutic effect of CAR-T may be altered by the dynamic changes of the intestinal microbiota^207^. Nevertheless, ongoing advances in elucidating underlying mechanisms continue to refine our comprehension of CLD pathogenesis, laying a solid foundation for the development of precision-medicine approaches to CLD.

## Conclusion

Over the past few years, the evidence that “leaky gut and gut dysbiosis contribute to the hepatic diseases pathogenesis” has sparked growing interest in the gut–liver axis. As the primary physical conduit linking the intestine and the liver, the gut–liver axis plays a central role in interconnecting gut microbiota and hepatic immunity. This axis harbours a diverse array of immune cells that are continually exposed to intestinal contents. Herein, we summarize how microbe-derived signals continuously shape T cell differentiation, function polarization and phenotypic plasticity. Reciprocally, T cells can modulate the composition and metabolic activity of intestinal microbiota. Studies spanning AILD, ALD, MASLD and viral hepatitis collectively underscore the remarkable diversity and complexity of microbiota–T cell interactions, highlighting a promising direction for CLD treatment. Strategies such as CAR-T therapy and autologous Treg cell transfer seem to have achieved initial efficacy in the treatment of AILD. Besides, probiotics, prebiotics and FMT also hold great potential as adjuvant therapies for liver disorders. However, the bidirectional and dynamic nature of the gut–liver–immune axis mandates cautious and patient-stratified approaches. Future research should focus on elucidating specific microbial and immunological mechanisms underlying disease pathogenesis, validating candidate therapeutic targets in clinical settings, and advancing towards precision-medicine paradigms for CLD management. A deeper integration of microbiome science with translational immunology will undoubtedly pave the way for more effective and sustainable interventions in CLD.

## Data Availability

Not applicable.
